# Nitrate of Silver as a Remedy for Burns

**Published:** 1856-03

**Authors:** John Wiltbank

**Affiliations:** Philadelphia


### *Nitrate of Silver as a Remedy for Burns.*

Dr. Hollingsworth :

*Dear Sir,*—I wish to call the attention of the readers of the Examiner to the value of the nitrate of silver as an application to burns and scalds. I have used it frequently both in deep and superficial burns, and I have been equally surprised and gratified by the results. The advantages of the caustic application are numerous. It furnishes a complete protection to the inflamed surface, subdues the pain, arrests the serous discharge, changes the character of the inflammation, promotes a speedy cure, and, if I am not mistaken, prevents the formation of those ugly cicatrices and the irregular contractions of the skin which so often occur in the healing of burns.

The mode of application is simple. In superficial burns a strong solution—20 to 40 grains of the nitrate to the ounce of water—should be applied over the whole surface with a camel's hair pencil, vesications should be opened and the surface carefully wiped dry before the solution is applied. If the burn is deep and the discharge of serum abundant, the entire surface of the ulcer should be touched lightly with the solid stick.

Very respectfully, John Wiltbank, M. D.

*Philadelphia, February 7th,* 1856.

